# Traumatic cervical spinal cord transection

**DOI:** 10.1259/bjrcr.20180043

**Published:** 2018-10-11

**Authors:** Mai A Mostafa

**Affiliations:** 1 Radiology Department, Ain-shams University, El Demerdash teaching Hospital, Abbassia, Egypt

## Abstract

MRI plays a crucial role in the assessment of spinal cord injury in cervical trauma. Transection of the cord is a rare post-traumatic cord injury, which appears on *T*_2_W images as a high signal between the two disrupted ends of the cord.

## History

A 20-year-old male patient presented with quadriplegia and urinary incontinence after a car accident. There was no relevant neurological deficit before the accident. Patient was admitted to ICU till he was discharged to professional nursing care provider house.

## Image findings

**Figure 1.**
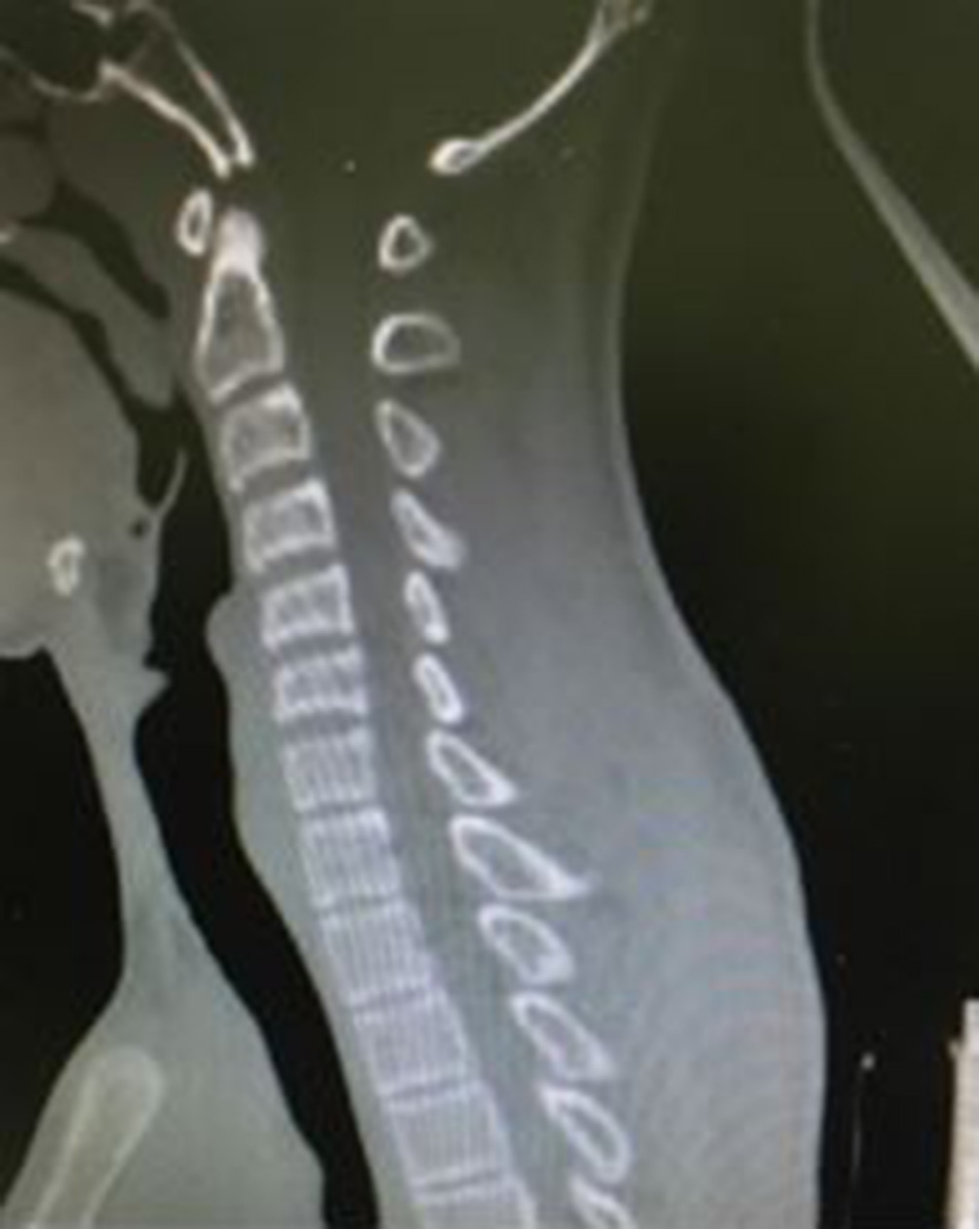
CT cervical spine sagittal reformatted image reveal straightening of cervical curvature.

**Figure 2.**
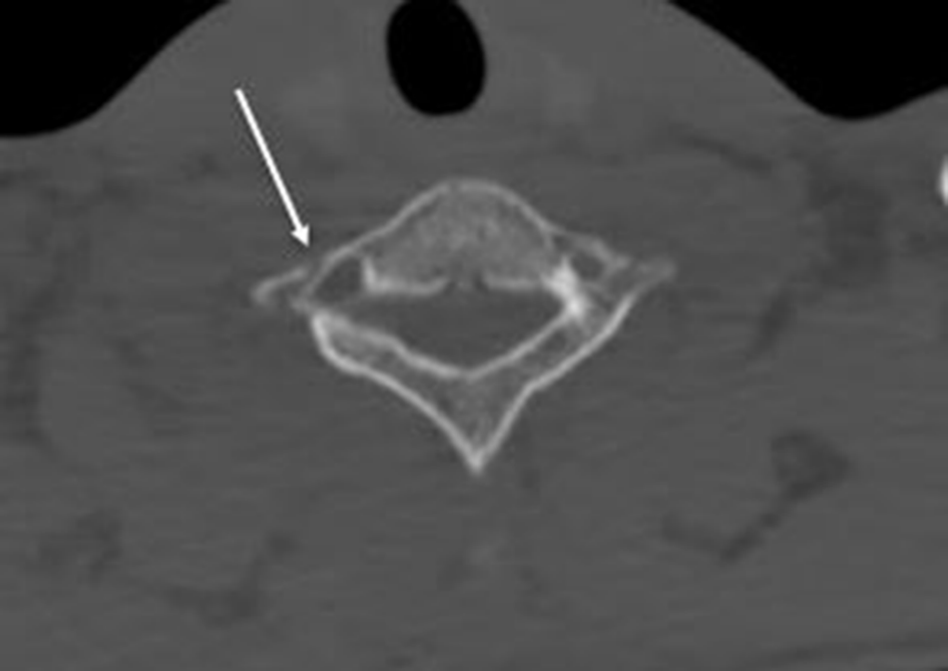
CT axial cut shows right fracture transverse process of C7.

**Figure 3.**
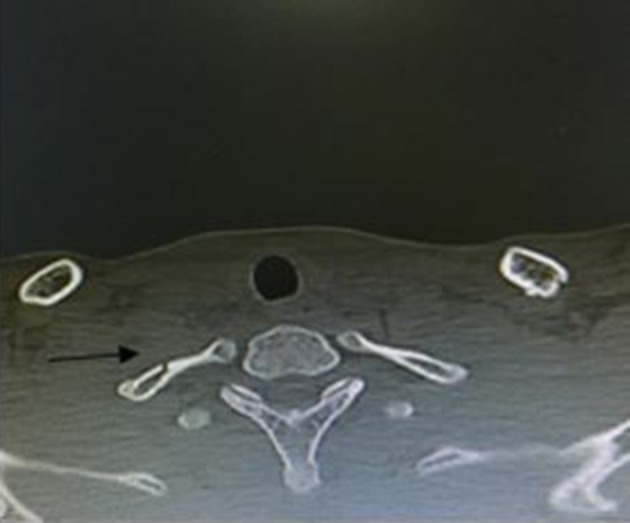
CT axial cut shows fracture in right first rib.

**Figure 4.**
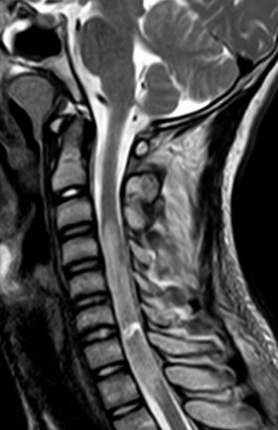
MRI sagittal *T*_2_WI shows C6-7 linear fluid signal denoting transection. *T*_2_W, *T*_2_ weighted imaging.

**Figure 5.**
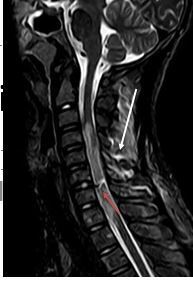
MRI sagittal STIR showsC6-7 linear fluid signal denoting transection (red arrow)with interspinous ligament injury (white arrow).

CT cervical spine revealed straightening of cervical curvature (Figure 1) and fracture in the right transverse process of C7 (Figure 2) and right first rib (Figure 3). There was not subluxation or dislocation. MRI images revealed C6-7 linear fluid signal intensity on *T*_2_ weighted imaging (*T*_2_ WI) (Figure 4) (*T*_1_ weighted imaging (*T*_1_WI) and short tau inversion-recovery (STIR). Figure 5 involves near total cervical cord circumference denoting cord transection. Cord oedema exhibits low signal intensity on *T*_1_WI and high signal intensity on *T*_2_WI & STIR, involving a long segment that extends from C3 to D1 with interspinous ligament injury and without concurrent epidural haematoma.

## Management of SCI

### Initial management

- Immediate resuscitation using the basic “ABC” principles.
- Spinal immobilization to avoid additional spinal damage.^1^

### Neuroprotection strategies

The aim of the treatment is to avoid hypoxia, hypotension and hypercarbia using vasopressor support and steroids.^1^

Surgery: Reduction by open or closed procedures, and surgical decompression should be considered to relieve the pressure on the cord.^1^

## Discussion

### Review of literature

- Spinal cord injury divided into traumatic and non-traumatic aetiology. Traumatic injuries occur due to external factors, as motor vehicle injury and sport-related injury, while non-traumatic occurs secondary to a disease process; such as a tumor, infection and degenerative disc disease.^2^
- Traumatic injuries are divided into phases, such as acute at first 48 h, subacute phase up to two weeks, intermediate phase within 6 months and chronic phase more than 6 months. The initial traumatic event usually causes mechanical disruption or dislocation of the vertebral column, which causes compression or transection of the spinal cord.^2^
- No reports of traumatic spinal cord transections in adults without fracture dislocation injuries in the sub axial spine are presented.^3^ In our case, no evidence of fracture dislocation or subluxation on CT and MR was mandatory to assess cord injury that would explain patient symptoms.

### Role of MRI in assessment of spinal cord injury^3^

- Spinal cord swelling.
- Spinal cord oedema, oedema length is proportionate to neurologic deficit and prognosis.
- Spinal cord haemorrhage.
- Cord compression.
- Cord transection.

## Learning points

1. Most patients with cord transaction require MRI for the detection of acute cord injury.
2. The role of MRI is to assess the injured cord, vertebral injury, disruption of ligaments and associated disc herniation.^4^
3. On sagittal *T*_2_ images, normal cord shows no signal abnormalities and oedema shows high signal intensity, while acute haemorrhage elicits hypointense signal with a thin rim of high signal intensity.^5^
4. The Ramon et al 18 study also involved different MRI signal patterns of compression, transaction and contusion. In compression, there will be a severe obliteration of the spinal cord, causing marked alteration of its morphology that prevent detection of abnormal signal, such as haemorrhage.
5. Transection shows discontinuity of the spinal cord on both *T*_1_WI and *T*_2_WI. Contusion shows normal images on *T*_1_WI, while the spinal cord on *T*_2_WI elicits a small central area of abnormal signal intensity.^5^
6. Epidural hematomas appear as extra axial collection of isointense to hyperintense signal on *T*_1_W images and hyperintense on *T*_2_W images.
7. STIR images detect oedema and ligamentous injuries, especially the interspinous or supraspinous ligaments. Although fat-suppressed *T*_2_W images detect edema, STIR images provide more fat suppression.^6^
